# Supplementary material for: Short-Term Joint Effects of PM10, NO2 and SO2 on Cardio-Respiratory Disease Hospital Admissions in Cape Town, South Africa
Source: Int J Environ Res Public Health. 2022 Jan 3;19(1):495. doi: 10.3390/ijerph19010495 (PMC8744938; doi:10.3390/ijerph19010495)
Supplement: Supplementary file 1 [file ijerph-19-00495-s001.zip › ijerph-1467725-supplementary.pdf]

Short-term joint effects of multiple air pollutants on cardio-respiratory disease hospital admissions in Cape Town, 2011 – 2016

Temitope Christina Adebayo-Ojo[1,2,] Janine Wichmann[3], Oluwaseyi Olalekan Arowosegbe[1,2], Nicole Probst-Hensch[1,2], Christian Schindler[1,2], Nino Künzli[1,2]

[1]Department of Epidemiology and Public Health, Swiss Tropical and Public Health Institute, Basel, Kreuzstrasse 2, 4123 Allschwil, Switzerland; oluwaseyiolalekan.arowosegbe@swisstph.ch (O.O.A.); nicole.probst@swisstph.ch (N.p.-H.); christian.schindler@swisstph.ch (C.S.); nino.kuenzli@swisstph.ch (N.K.)

[2]Faculty of Medicine, University of Basel, Basel, 4056, Switzerland

[3]Faculty of Health Sciences, School of Health Systems and Public Health, University of Pretoria, Pretoria, 0002, South Africa, janine.wichmann@up.ac.za
*Corresponding: temitope.adebayo@swisstph.ch

Supplentary material - Imputation analysis of ambient air pollutant data and time-series results

Hourly air pollution data for PM10, NO2 and SO2 were collected from 12 stations – seven stations for PM10, 12 for NO2 and SO2, respectively. These concentration were then aggregated to daily level provided at least 75% of the data for that day was available.

The figures below shows the annual proportion of daily means data available for each pollutant from the air quality monitoring stations.

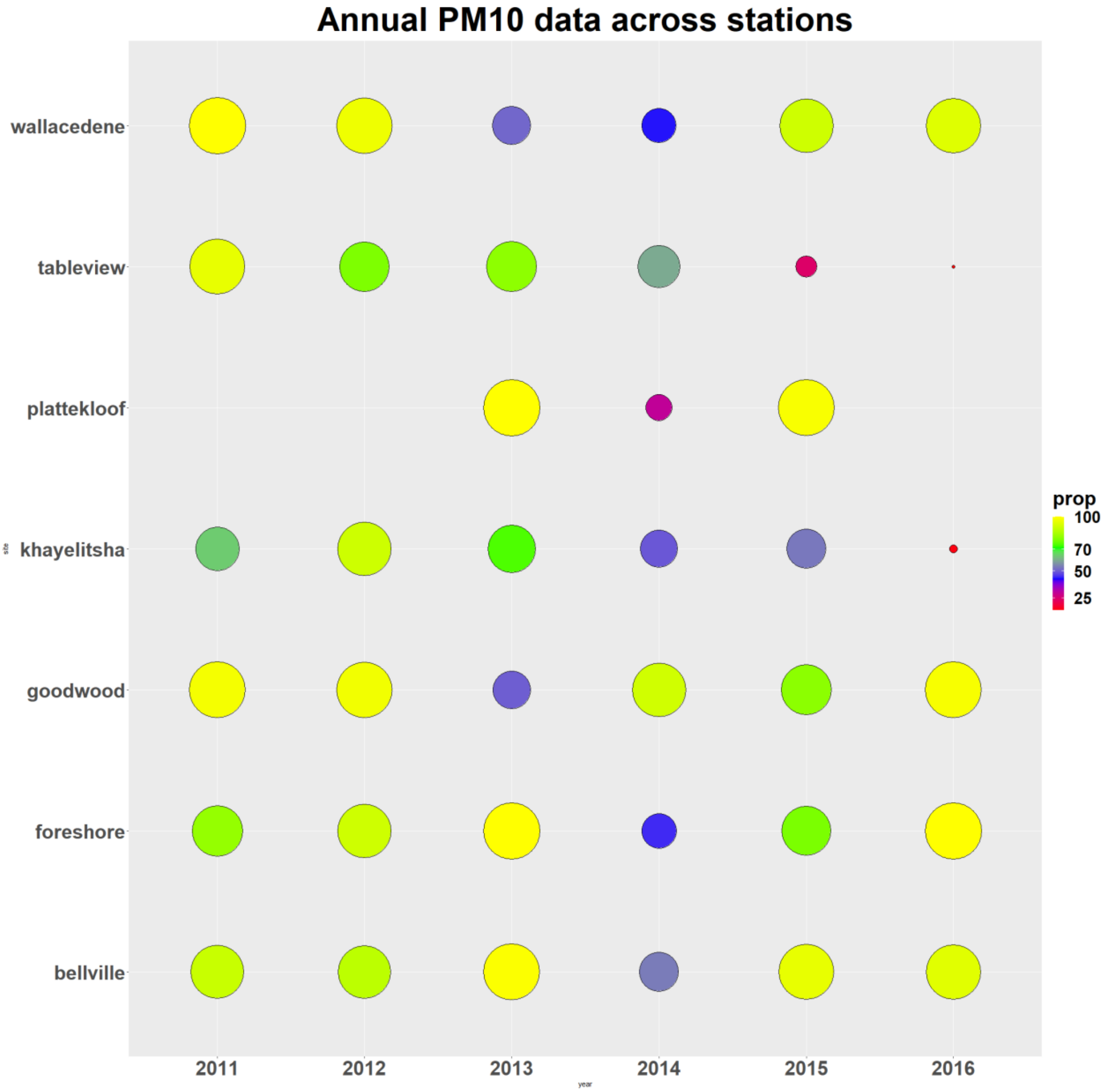

Figure S1: The proportion of PM10 daily means data collected from seven stations from 1st Jan 2011 – 31 October 2016 in Cape Town.

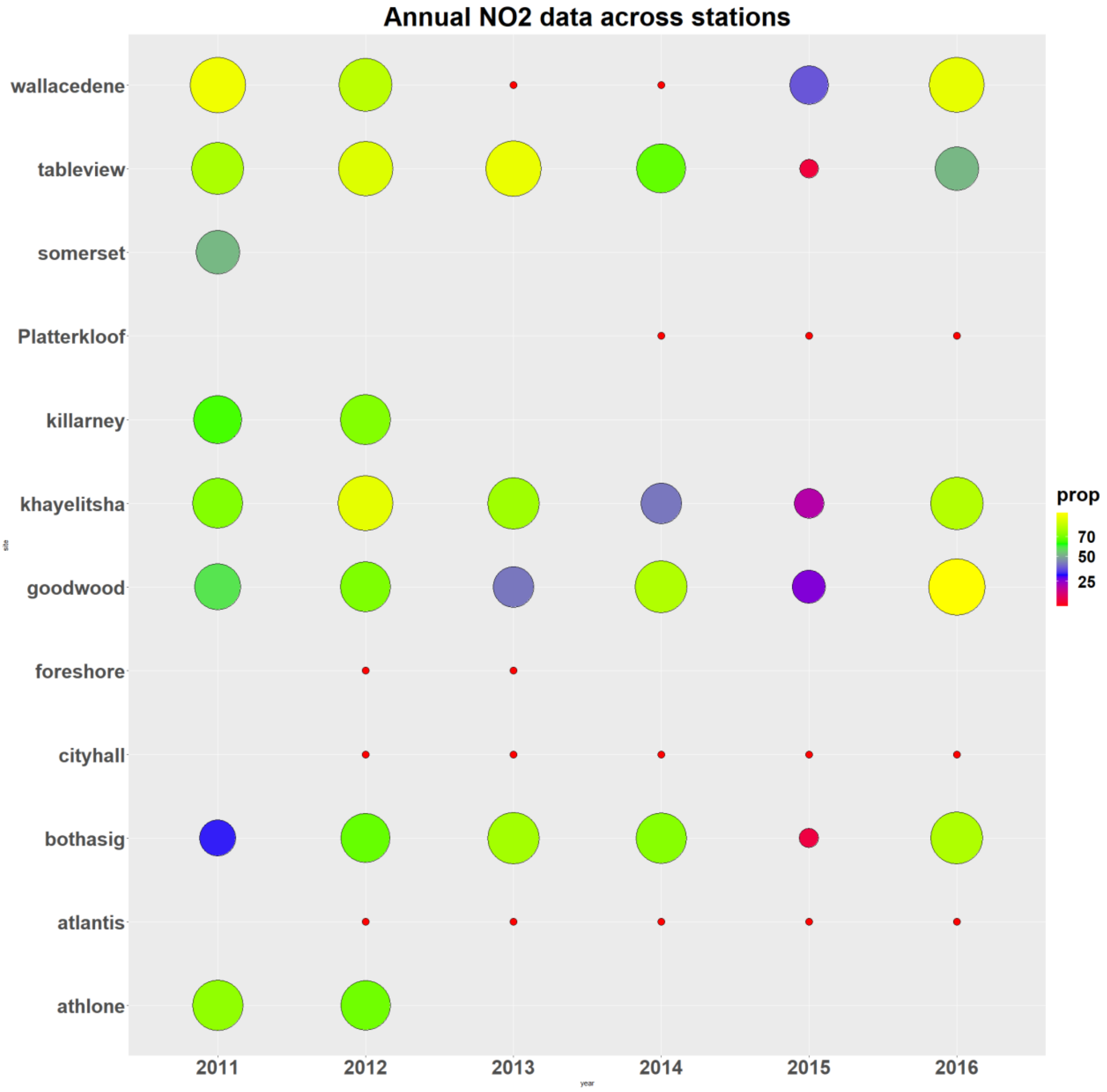

Figure S2: The proportion of NO2 daily means data collected from 12 stations from 1st Jan 2011 – 31 October 2016 in Cape Town.

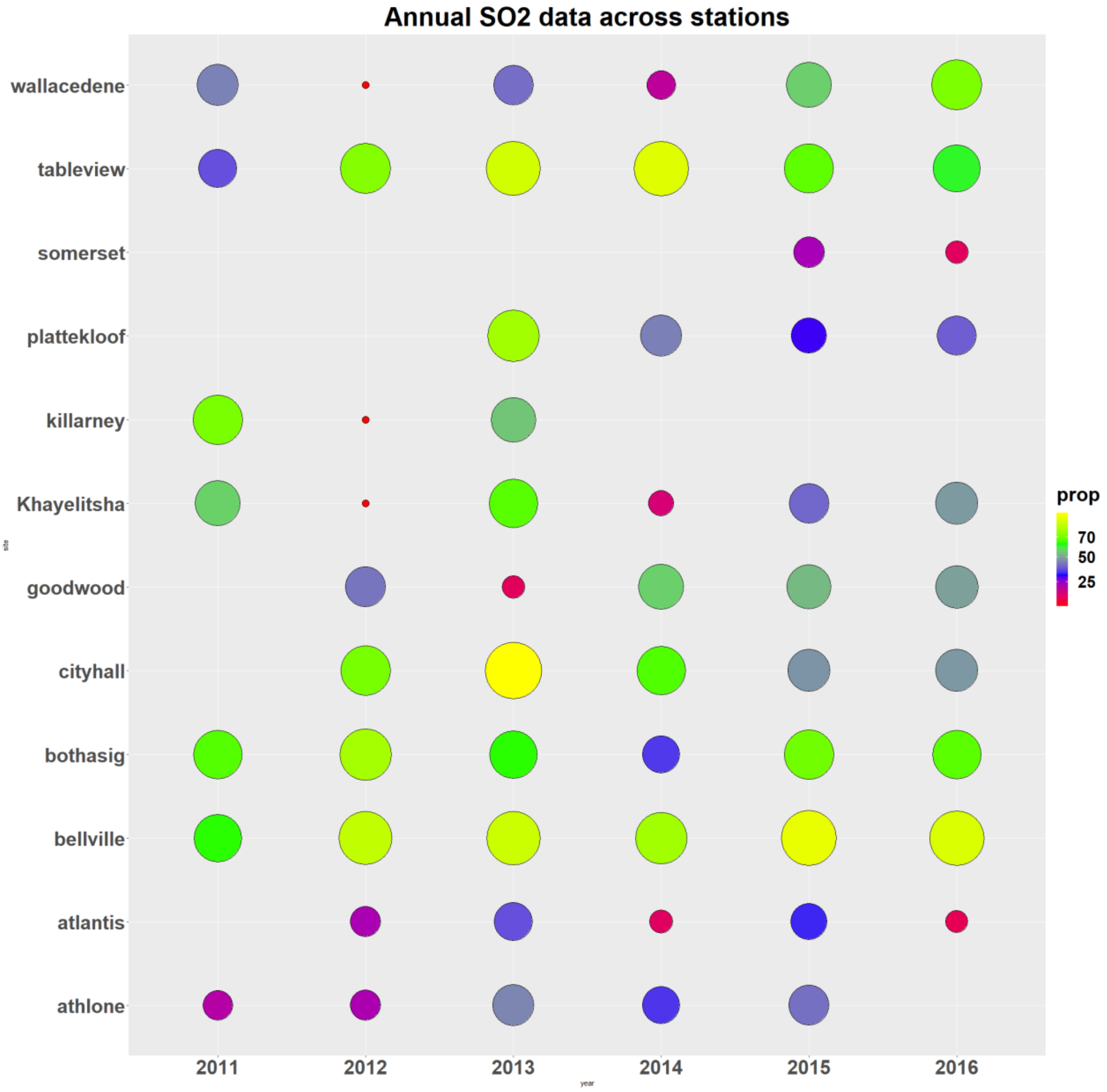

Figure S3: The proportion of SO2 daily means data collected from 12 stations from 1st Jan 2011 – 31 October 2016 in Cape Town.

The geographical locations of some of the stations and their classifications are shown in the map below.

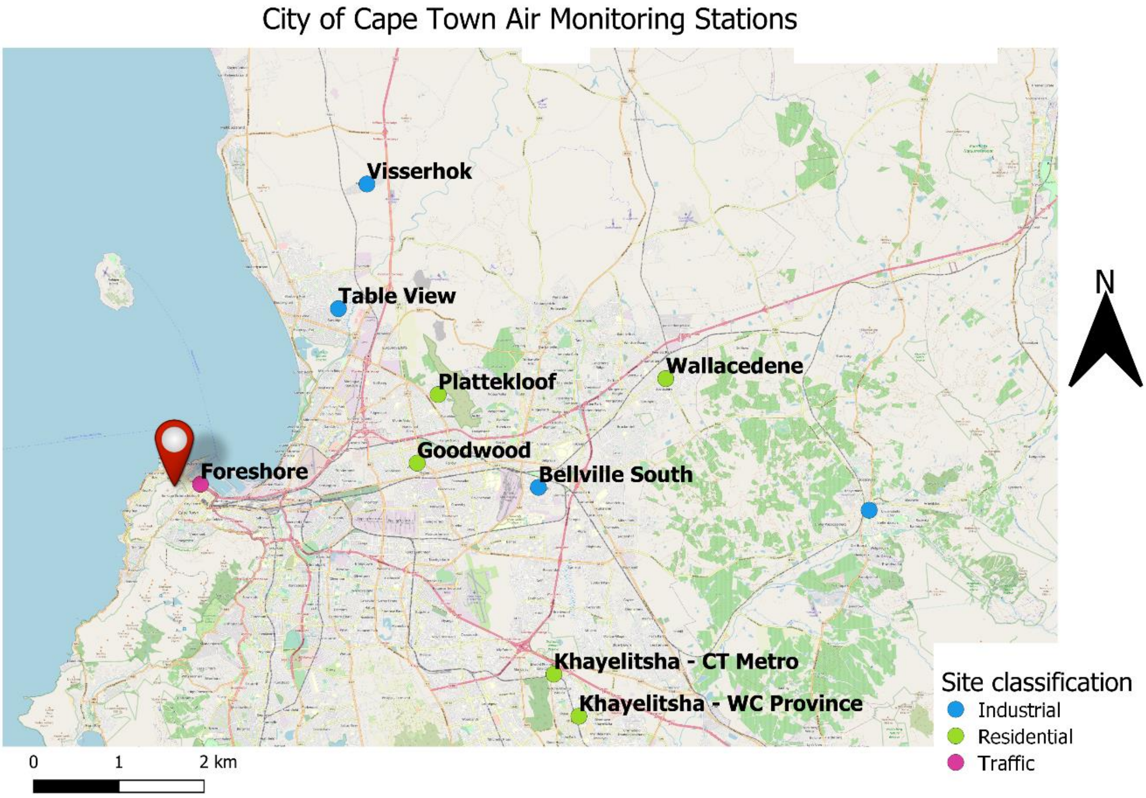

Figure S4: Air quality monitoring station in the City of Cape Town and their classifications. Stations shown on this map are those in close proximity to the City of Cape Town but all 12 stations were used in the analysis.

Correlation among stations

Spearman correlation was used to test the correlation among the stations for each pollutant's daily means during the the study period. We found high degree of correlation among PM10 stations, moderate correlation for NO2 and low degree of correlation among SO2 stations. This is illustrated in the correlation matrix below.

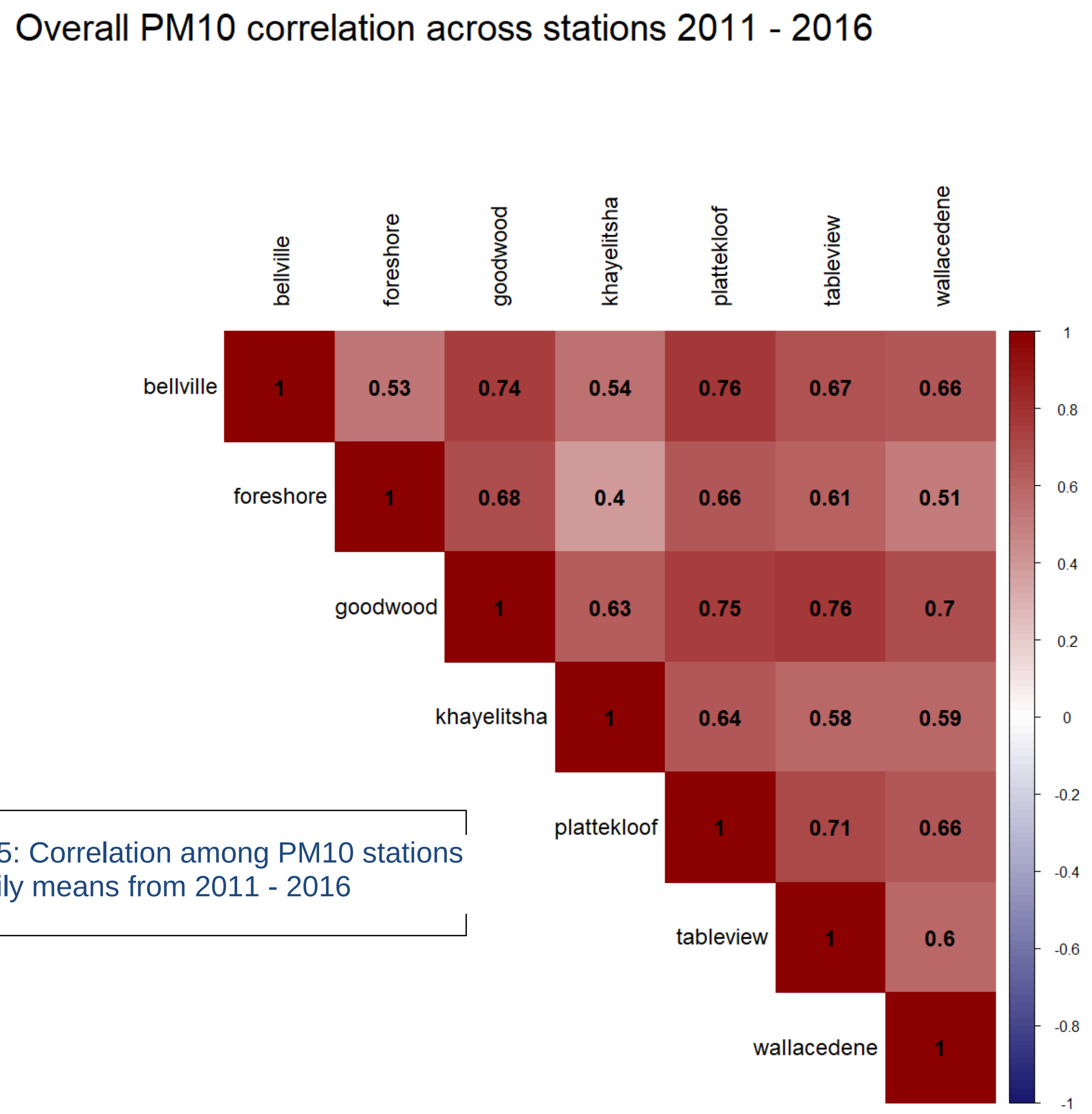

Figure S5: Correlation among PM10 stations using daily means from 2011 - 2016

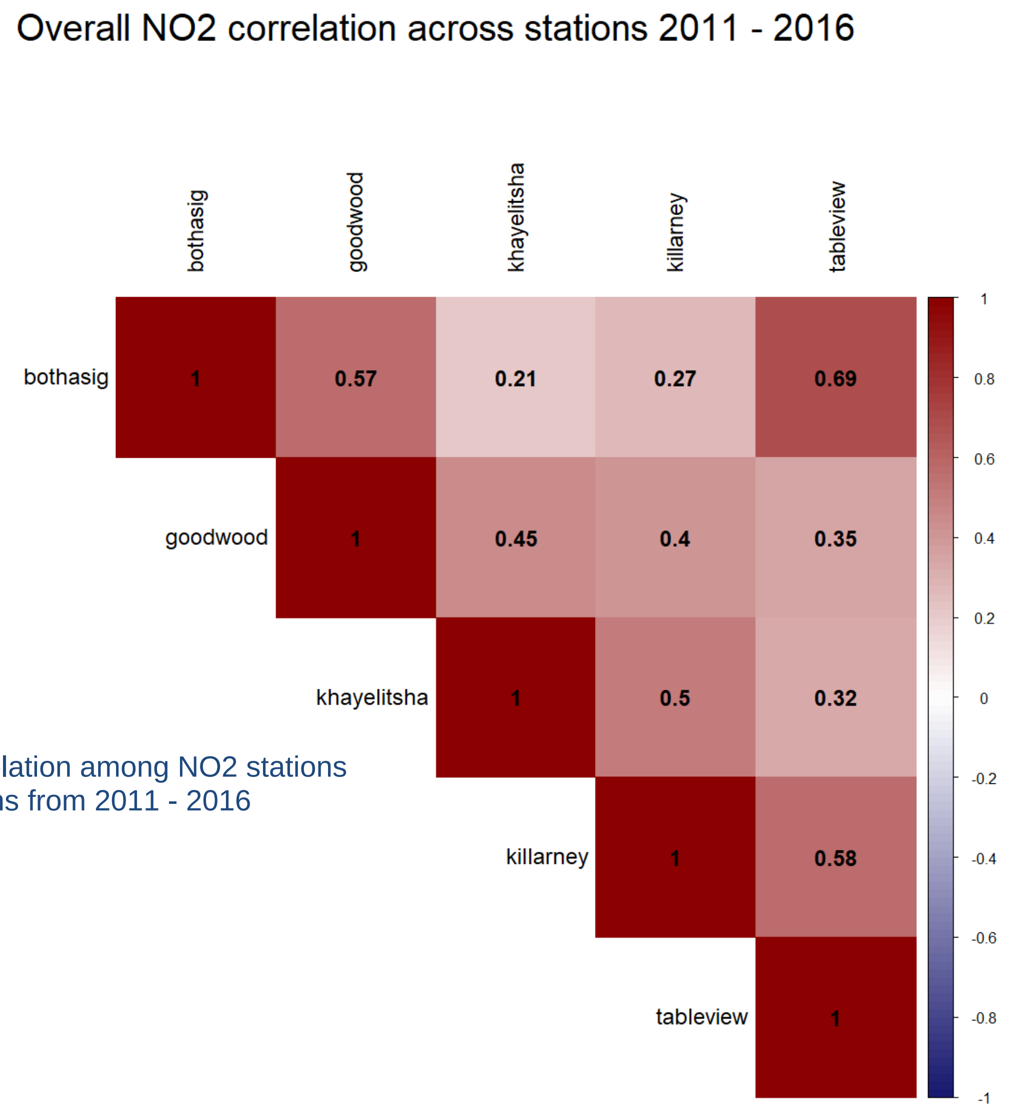

Figure S6: Correlation among NO2 stations using daily means from 2011 - 2016

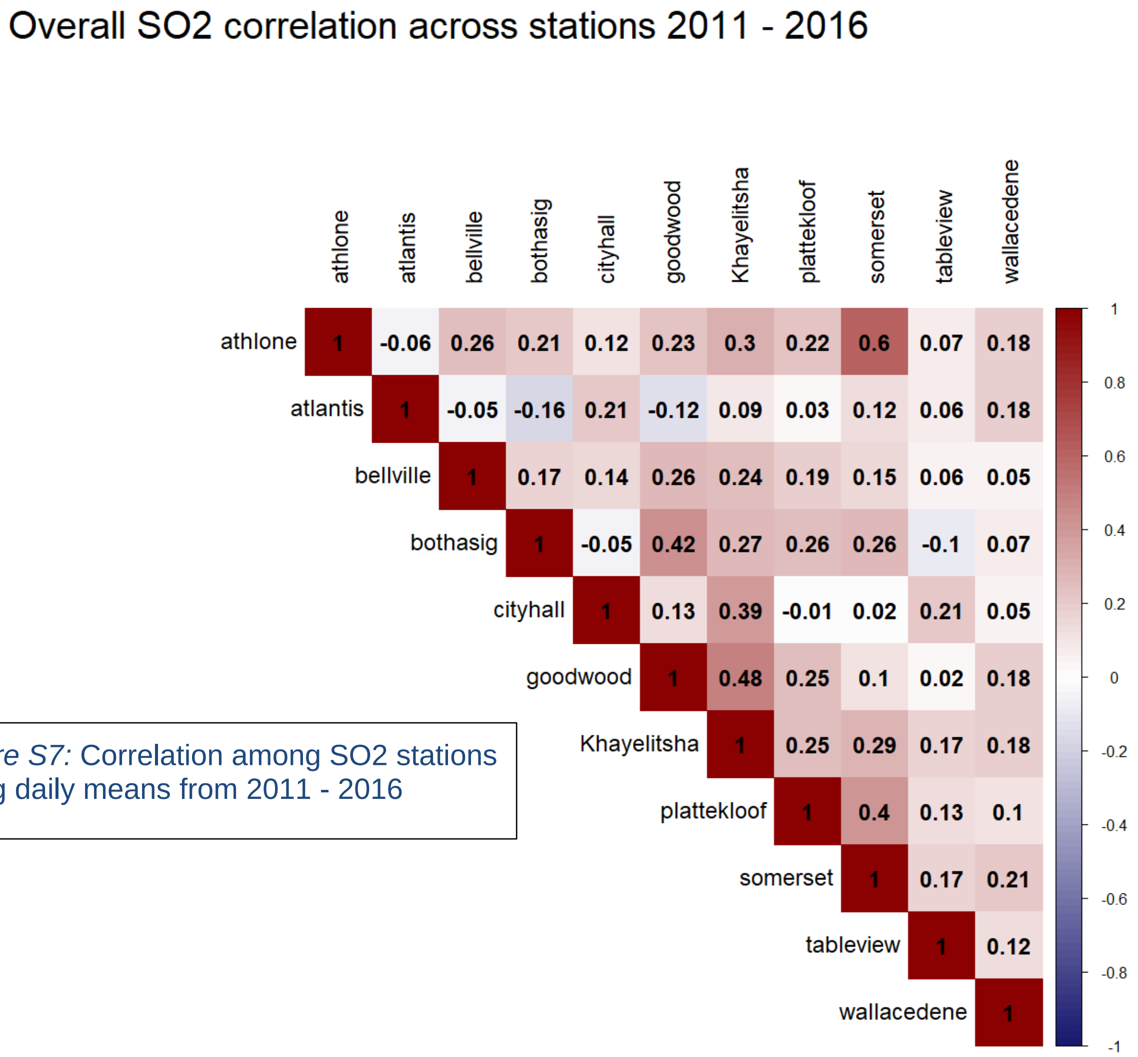

Figure S7: Correlation among SO2 stations using daily means from 2011 - 2016

Imputation of daily air pollutant data

The data obtained included missing days for all stations and pollutants; this is illustrated in *Figure S1Figure S1 Figure S2* and *Figure S3*. Thus, the intuitive solution for each pollutant was to take the average measurement on each day for all the stations with available data to obtain a city-level mean for the time-series analysis. However, this will introduce data gaps in the daily mean levels of the time series data. Therefore, we went a step further by imputing for stations with missing daily means we imputed an estimated mean based on the data of other stations. In case no station had a valid daily mean value, the concentration was defined as "missing". For instance, if day 5 has a daily city-level mean (that is mean across all stations) but 2 out of 7 stations had no measurement, we imputed daily means for those 2 stations. Furthermore, if day 10 has no measurement from all stations which means no city-level mean, this was left as missing and no imputation was done.

First we describe the notation as - Let $\mathcal{M} = \{1, \ldots, M\}$ denote the set of monitoring stations and let $C_i(t)$ denote the mean concentration of the pollutant of interest at station $i \; on \; day \; t$. Moreover, let $m_i(t)$ take the value 1 if a mean value is not available from station $i \; on \; day \; t$ and the value 0 otherwise.

Therefore,

$$S(t) = \sum_{i \in \mathcal{M} : m_i(t) = 0} C_{i\,(t)} \qquad (1)$$

The algorithm

In order to derive the daily city-level imputation factors, $S(t)$ is estimated using a non-linear regression model.

$$\begin{aligned} \mathrm{E}[S(t)] = \exp(&\beta_0 + \beta_1 t + \beta_2 t^2 + \beta_3 \cos(t/365.25 \times 2\pi) \\ &+ \beta_4 \sin(t/365.25 \times 2\pi) + \sum_{i \in \mathcal{M}} \gamma_i m_i(t) \\ &+ f(meteo\ variables) \\ &+ g(meteo\ variables, m_i(t), \ldots, m_M(t))) \end{aligned} \qquad (2)$$

The meteo variables used were temperature, relative humidity, wind speed and wind direction.

Where f is a linear function of meteo-variables with unknown parameters and g is a linear function of the product terms between meteo variables and missing indicators variables $m_i(t)$, each with an unknown parameter.

Furthermore, let

$$\begin{aligned} I(t) = \exp(&\beta_0 + \beta_1 t + \beta_2 t^2 + \beta_3 \cos(t/365.25 \times 2\pi) \\ &+ \beta_4 \sin(t/365.25 \times 2\pi) + \hat{f}(meteo\ variables)) \end{aligned} \qquad (3)$$

We then further defined

$$S^{imp}(t) = S(t)/\hat{S}(t) \times I(t) \quad (4)$$

Where $\hat{S}(t)$ is the estimate of $S(t)$ from the regression model. Finally, let

$$C(t) = \frac{S^{imp}(t)}{M} \quad (5)$$

If there are no missing values on a day t across all M stations, then all $m_i(t)$ are 0, implying that $I(t) = \hat{S}(t)$ and thus $S^{imp}(t) = S(t)$. In this case, $C(t) = \frac{S(t)}{M}$ equal the mean of daily concentration values across all M stations. Therefore, on days with complete data, the algorithm makes no imputation.

Time-series analysis results.

Table S1: Temporal spearman rank correlation coefficients among daily mean concentrations of ambient air pollutants and meteorological variables during the year and by seasons

	PM_{10}	NO_2	SO_2	Temperature	Humidity
	Overall year				
PM_{10}	1				
NO_2	0.302	1			
SO_2	0.195	0.274	1		
Temperature	0.227	-0.378	0.006	1	
Humidity	-0.293	0.019	-0.108	-0.392	1
Warm season	September – April				
PM10	1				
NO2	0.19	1			
SO2	0.174	0.184	1		
Temperature	0.391	-0.214	0.065	1	
Humidity	-0.303	-0.038	-0.126	-0.234	1
Cold season	May – August				
PM_{10}	1				
NO_2	0.572	1			
SO_2	0.321	0.402	1		
Temperature	0.026	-0.087	0.067	1	
Humidity	-0.271	-0.337	-0.231	-0.006	1

Table S2: Single and multiple pollutant model adjusted relative risk (RR) for an interquartile range increase in the two-day moving average of PM10 concentrations and hospital admissions due to cardiovascular and respiratory diseases in all ages, age groups and sex, Cape Town, South Africa, 1 January 2011 - 31 October.

		Respiratory disease			Cardiovascular disease		
	Pollutant IQR (12ug/m3)	Relative risk	95% Confidence interval		Relative risk	95% Confidence interval	
			Lower interval	Upper interval		Lower interval	Upper interval
All ages and sex	PM10	1.019	1.005	1.032	1.021	1.006	1.035
Age 0 - 14		1.02	1.002	1.039			
Age 15 - 64		1.009	0.988	1.03	1.021	1.002	1.039
Age >65		1.019	0.994	1.046	1.022	1.004	1.041
Female		1.014	0.997	1.032	1.02	1.001	1.04
Male		1.02	1.002	1.037	1.021	1.003	1.038
All ages and sex	$PM10_{NO2}$	1.009	0.992	1.026	1.02	1.003	1.038
Age 0 - 14		1.008	0.984	1.031			
Age 15 - 64		1.005	0.978	1.033	1.022	1	1.045
Age >65		1.02	0.986	1.055	1.022	0.999	1.044
Female		1.006	0.985	1.029	1.022	0.997	1.046
Male		1.012	0.989	1.034	1.02	0.999	1.042
All ages and sex	$PM10_{SO2}$	1.017	1.003	1.031	1.022	1.007	1.037
Age 0 - 14		1.016	0.997	1.035			
Age 15 - 64		1.011	0.989	1.033	1.021	1.002	1.041
Age >65		1.023	0.995	1.051	1.026	1.007	1.045
Female		1.016	0.998	1.034	1.02	0.999	1.041
Male		1.016	0.998	1.035	1.026	1.008	1.044

All ages and sex	$PM10_{NO2\ SO2}$	1.008	0.991	1.026	1.021	1.002	1.039
Age 0 - 14		1.004	0.98	1.028			
Age 15 - 64		1.003	0.975	1.031	1.02	0.997	1.044
Age >65		1.024	0.989	1.06	1.025	1.002	1.048
Female		1.006	0.984	1.029	1.02	0.996	1.046
Male		1.01	0.987	1.033	1.024	1.002	1.046

Interquartile range: PM10: 12 ug/m3, NO2 7.3 µg/m3, SO2 = 3.6 µg/m3. Relative risk estimated from Quasi-Poison regression models, adjusting for time trends and seasonal variations, day of week, holiday meteorological factors including temperature and relative humidity. $PM10_{N02}$, $PM10_{SO2}$ and $PM10_{NO2\ SO2}$ refers to the estimate of PM10 in multiple pollutant models. For cardiovascular disease age 0-14 was excluded from the modelling due to small sample size.

Table S3: Single and multiple pollutant model adjusted relative risk (RR) for an interquartile range increase in the two-day moving average of NO2 concentrations and hospital admissions due to cardiovascular and respiratory diseases in all ages, age groups and sex, Cape Town, South Africa, 1 January 2011 - 31 October.

		Respiratory disease			Cardiovascular disease		
	Pollutant IQR (7.3 ug/m3)	Relative risk	95% Confidence interval		Relative risk	95% Confidence interval	
			Lower interval	Upper interval		Lower interval	Upper interval
All ages and sex	NO2	1.023	1.006	1.04	1.01	0.992	1.028
Age 0 - 14		1.031	1.007	1.056			
Age 15 - 64		1.003	0.976	1.03	1.006	0.983	1.03
Age >65		1.005	0.972	1.039	1.017	0.994	1.041
Female		1.013	0.991	1.036	1.007	0.983	1.033
Male		1.019	0.997	1.042	1.015	0.993	1.037

All ages and sex		1.016	0.995	1.038	0.997	0.976	1.019
Age 0 - 14		1.027	0.998	1.058			
Age 15 - 64	$NO2_{PM10}$	1.012	0.979	1.047	0.992	0.965	1.02
Age >65		0.995	0.953	1.038	1.003	0.977	1.031
Female		1.018	0.99	1.047	0.994	0.965	1.024
Male		1.012	0.985	1.041	1.002	0.976	1.028
All ages and sex		1.021	1.003	1.039	1.013	0.993	1.032
Age 0 - 14		1.032	1.007	1.058			
Age 15 - 64	$NO2_{SO2}$	1.01	0.981	1.039	1.009	0.985	1.033
Age >65		1.015	0.979	1.053	1.02	0.997	1.045
Female		1.021	0.998	1.045	1.006	0.981	1.033
Male		1.018	0.995	1.042	1.019	0.997	1.043
All ages and sex		1.015	0.994	1.037	0.999	0.977	1.022
Age 0 - 14		1.029	0.999	1.061			
Age 15 - 64	$NO2_{PM10\ SO2}$	1.009	0.975	1.044	0.996	0.969	1.024
Age >65		0.999	0.956	1.043	1.005	0.978	1.033
Female		1.017	0.989	1.046	0.994	0.965	1.024
Male		1.011	0.983	1.04	1.005	0.979	1.032

Interquartile range: PM10: 12 ug/m3, NO2 7.3 μg/m3, SO2 = 3.6 μg/m3. Relative risk estimated from Quasi-Poison regression models, adjusting for time trends and seasonal variations, day of week, holiday meteorological factors including temperature and relative humidity. $NO2_{PM10}$, $NO2_{SO2}$ and $NO2_{PM10\ SO2}$ refers to the estimate of NO2 in multiple pollutant models. For cardiovascular disease age 0-14 was excluded from the modelling due to small sample size.

Table S4: Single and multiple pollutant model adjusted relative risk (RR) for an interquartile range increase in the two-day moving average of SO2 concentrations and hospital admissions due to cardiovascular and respiratory diseases in all ages, age groups and sex, Cape Town, South Africa, 1 January 2011 - 31 October.

		Respiratory disease			Cardiovascular disease		
	Pollutant IQR (3.2ug/m3)	Relative risk	95% Confidence interval		Relative risk	95% Confidence interval	
			Lower interval	Upper interval		Lower interval	Upper interval
All ages and sex	SO2	1.011	0.998	1.024	0.997	0.984	1.011
Age 0 - 14		1.015	0.997	1.033			
Age 15 - 64		1.011	0.99	1.032	0.998	0.98	1.015
Age >65		0.989	0.963	1.015	0.995	0.978	1.012
Female		1.006	0.989	1.023	1.009	0.99	1.028
Male		1.015	0.998	1.032	0.987	0.971	1.003
All ages and sex	$SO2_{PM10}$	1.006	0.993	1.02	0.993	0.979	1.007
Age 0 - 14		1.011	0.991	1.03			
Age 15 - 64		1.011	0.989	1.033	0.992	0.974	1.011
Age >65		0.986	0.959	1.014	0.989	0.972	1.007
Female		1.005	0.987	1.023	1.004	0.985	1.024
Male		1.008	0.99	1.027	0.981	0.965	0.998
All ages and sex	$SO2_{NO2}$	1.005	0.991	1.019	0.999	0.984	1.014
Age 0 - 14		1.005	0.985	1.025			
Age 15 - 64		1.016	0.992	1.039	1.001	0.982	1.02
Age >65		0.981	0.953	1.011	0.992	0.974	1.011
Female		1.003	0.984	1.022	1.008	0.987	1.028
Male		1.007	0.988	1.026	0.987	0.969	1.004
All ages and sex	$SO2_{PM10\ NO2}$	1.003	0.989	1.018	0.996	0.981	1.011

Age 0 - 14		1.004	0.984	1.025			
Age 15 - 64		1.015	0.991	1.039	0.998	0.979	1.017
Age >65		0.978	0.949	1.008	0.989	0.97	1.008
Female		1.001	0.982	1.021	1.004	0.984	1.025
Male		1.006	0.986	1.025	0.983	0.965	1.001

Interquartile range: PM10: 12 ug/m3, NO2 7.3 µg/m3, SO2 = 3.6 µg/m3. Relative risk estimated from Quasi-Poison regression models, adjusting for time trends and seasonal variations, day of week, holiday meteorological factors including temperature and relative humidity. $SO2_{PM10}$, $SO2_{NO2}$ and $SO2_{PM10\ NO2}$ refers to the estimate of SO2 in multiple pollutant models. For cardiovascular disease age 0-14 was excluded from the modelling due to small sample size. For cardiovascular disease age 0-14 was excluded from the modelling due to small sample size.

Table S5: Warm season – Single and multiple pollutant model adjusted relative risk (RR) for an interquartile range increase in the two-day moving average of PM10 concentrations and hospital admissions due to cardiovascular and respiratory diseases in all ages, Cape Town, South Africa, 1 January 2011 - 31 October.

		Respiratory disease			Cardiovascular disease		
	Pollutant IQR (ug/m3)	Relative risk	95% Confidence interval		Relative risk	95% Confidence interval	
			Lower interval	Upper interval		Lower interval	Upper interval
All ages and sex	PM10	1.022	1.005	1.039	1.034	1.015	1.053
	NO2	1.013	0.994	1.033	1.006	0.988	1.025
	SO2	1.023	0.996	1.049	1.027	1.002	1.054

Interquartile range: PM10: 9.7 ug/m3, NO2 5.4 µg/m3, SO2 = 3.0 µg/m3. Relative risk estimated from Quasi-Poison regression models, adjusting for time trends and seasonal variations, day of week, holiday meteorological factors including temperature and relative humidity. $PM10_{N02}$, $PM10_{SO2}$ and $PM10_{NO2\ SO2}$ refers to the estimate of PM10 in multiple pollutant models. For cardiovascular disease age 0-14 was excluded from the modelling due to small sample size. The warm season were the months of September – April.

Table S6: Cold season – Single and multiple pollutant model adjusted relative risk (RR) for an interquartile range increase in the two-day moving average of PM10 concentrations and hospital admissions due to cardiovascular and respiratory diseases in all ages, Cape Town, South Africa, 1 January 2011 - 31 October.

		Respiratory disease			Cardiovascular disease		
	Pollutant IQR (ug/m3)	Relative risk	95% Confidence interval		Relative risk	95% Confidence interval	
			Lower interval	Upper interval		Lower interval	Upper interval
All ages and sex	PM10	1.013	0.994	1.033	1.006	0.988	1.025
	NO2	1.023	0.996	1.049	1.027	1.002	1.054
	SO2	1.023	1.001	1.046	1	0.977	1.023

Table S7: Single and multiple pollutant model adjusted relative risk (RR) for 10 ug/m3 increase in the two-day moving average of PM10 concentrations and hospital admissions due to cardiovascular and respiratory diseases in all ages, age groups and sex, Cape Town, South Africa, 1 January 2011 - 31 October.

		Respiratory disease			Cardiovascular disease		
	Pollutant (10ug/m3)	Relative risk	95% Confidence interval		Relative risk	95% Confidence interval	
			Lower interval	Upper interval		Lower interval	Upper interval
All ages and sex	PM10	1.018	1.005	1.03	1.019	1.006	1.033
Age 0 - 14		1.02	1.002	1.037			
Age 15 - 64		1.009	0.989	1.029	1.019	1.001	1.037
Age >65		1.018	0.994	1.044	1.021	1.004	1.039
Female		1.014	0.997	1.031	1.019	1.001	1.039
Male		1.019	1.002	1.035	1.02	1.003	1.036
All ages and sex	$PM10_{NO2}$	1.009	0.993	1.025	1.019	1.002	1.036
Age 0 - 14		1.007	0.985	1.03			
Age 15 - 64		1.005	0.979	1.031	1.021	1	1.043
Age >65		1.019	0.986	1.052	1.021	0.999	1.042
Female		1.006	0.985	1.027	1.021	0.998	1.044
Male		1.011	0.99	1.033	1.019	0.999	1.04
All ages and sex	$PM10_{SO2}$	1.016	1.003	1.029	1.023	1.009	1.038
Age 0 - 14		1.018	0.999	1.036			
Age 15 - 64		1.01	0.989	1.032	1.02	1.002	1.039
Age >65		1.022	0.995	1.049	1.025	1.007	1.043
Female		1.015	0.998	1.033	1.019	0.999	1.039
Male		1.016	0.998	1.033	1.025	1.008	1.042
All ages and sex	$PM10_{NO2\ SO2}$	1.008	0.992	1.025	1.022	1.005	1.039

Age 0 - 14		1.006	0.983	1.029			
Age 15 - 64		1.003	0.976	1.029	1.021	0.999	1.043
Age >65		1.023	0.99	1.057	1.024	1.002	1.046
Female		1.006	0.985	1.028	1.019	0.996	1.044
Male		1.01	0.988	1.032	1.023	1.002	1.044

Average: PM10: 24.4 ug/m3, NO2 15 µg/m3, SO2 = 9.4 µg/m3. Relative risk estimated from Quasi-Poison regression models, adjusting for time trends and seasonal variations, day of week, holiday meteorological factors including temperature and relative humidity. $PM10_{N02}$, $PM10_{SO2}$ and $PM10_{NO2\ SO2}$ refers to the estimate of PM10 in multiple pollutant models. For cardiovascular disease age 0-14 was excluded from the modelling due to small sample size.

Table S8: Single and multiple pollutant model adjusted relative risk (RR) for 10 ug/m3 increase in the two-day moving average of NO2 concentrations and hospital admissions due to cardiovascular and respiratory diseases in all ages, age groups and sex, Cape Town, South Africa, 1 January 2011 - 31 October.

		Respiratory disease			Cardiovascular disease		
	Pollutant IQR (6.8ug/m3)	Relative risk	95% Confidence interval		Relative risk	95% Confidence interval	
			Lower interval	Upper interval		Lower interval	Upper interval
All ages and sex	NO2	1.034	1.008	1.059	1.013	0.987	1.04
Age 0 - 14		1.048	1.013	1.084			
Age 15 - 64		1.004	0.965	1.044	1.009	0.975	1.044
Age >65		1.008	0.959	1.058	1.025	0.991	1.06
Female		1.021	0.989	1.055	1.011	0.975	1.049
Male		1.029	0.996	1.062	1.022	0.989	1.055
All ages and sex	$NO2_{PM10}$	1.024	0.993	1.056	0.996	0.964	1.028
Age 0 - 14		1.04	0.997	1.086			
Age 15 - 64		1.018	0.969	1.07	0.988	0.949	1.029

Age >65		0.992	0.931	1.057	1.005	0.966	1.046
Female		1.027	0.986	1.069	0.991	0.949	1.035
Male		1.018	0.978	1.061	1.003	0.965	1.041
All ages and sex	$NO2_{SO2}$	1.031	1.004	1.058	1.022	0.994	1.051
Age 0 - 14		1.045	1.008	1.084			
Age 15 - 64		1.015	0.973	1.058	1.01	0.976	1.047
Age >65		1.023	0.97	1.079	1.03	0.995	1.066
Female		1.032	0.997	1.067	1.009	0.972	1.048
Male		1.027	0.992	1.063	1.029	0.995	1.063
All ages and sex	$NO2_{PM10\ SO2}$	1.022	0.991	1.054	1.001	0.969	1.034
Age 0 - 14		1.039	0.995	1.085			
Age 15 - 64		1.013	0.963	1.065	0.992	0.952	1.033
Age >65		0.998	0.936	1.064	1.007	0.968	1.049
Female		1.026	0.984	1.069	0.991	0.949	1.036
Male		1.016	0.975	1.059	1.008	0.97	1.047

Average: PM10: 24.4 ug/m3, NO2 15 μg/m3, SO2 = 9.4 μg/m3. Relative risk estimated from Quasi-Poison regression models, adjusting for time trends and seasonal variations, day of week, holiday meteorological factors including temperature and relative humidity. $NO2_{PM10}$, $NO2_{SO2}$ and $NO2_{PM10\ SO2}$ refers to the estimate of NO2 in multiple pollutant models. For cardiovascular disease age 0-14 was excluded from the modelling due to small sample size.

Table S9: Single and multiple pollutant model adjusted relative risk (RR) for 10 ug/m3 increase in the two-day moving average of SO2 concentrations and hospital admissions due to cardiovascular and respiratory diseases in all ages, age groups and sex, Cape Town, South Africa, 1 January 2011 - 31 October.

		Respiratory disease			Cardiovascular disease		
	Pollutant IQR (3.2ug/m3)	Relative risk	95% Confidence interval		Relative risk	95% Confidence interval	
			Lower interval	Upper interval		Lower interval	Upper interval
All ages and sex	SO2	1.035	0.993	1.078	0.988	0.947	1.031
Age 0 - 14		1.044	0.988	1.104			
Age 15 - 64		1.035	0.97	1.103	0.993	0.94	1.049
Age >65		0.966	0.891	1.048	0.984	0.932	1.039
Female		1.012	0.96	1.067	1.028	0.97	1.091
Male		1.035	0.978	1.094	0.96	0.912	1.011
All ages and sex	$SO2_{PM10}$	1.02	0.977	1.064	0.973	0.931	1.017
Age 0 - 14		1.03	0.971	1.093			
Age 15 - 64		1.034	0.965	1.107	0.976	0.923	1.033
Age >65		0.958	0.878	1.046	0.967	0.914	1.023
Female		1.015	0.96	1.073	1.014	0.954	1.077
Male		1.025	0.969	1.085	0.943	0.894	0.994
All ages and sex	$SO2_{NO2}$	1.015	0.971	1.062	0.985	0.94	1.032
Age 0 - 14		1.016	0.954	1.081			
Age 15 - 64		1.05	0.977	1.128	0.997	0.941	1.058
Age >65		0.943	0.86	1.034	0.976	0.92	1.035
Female		1.01	0.952	1.071	1.024	0.961	1.091
Male		1.021	0.962	1.084	0.959	0.907	1.014
All ages and sex	$SO2_{PM10\ NO2}$	1.01	0.965	1.057	0.975	0.931	1.022

Age 0 - 14		1.012	0.95	1.078			
Age 15 - 64		1.046	0.973	1.126	0.986	0.929	1.046
Age >65		0.934	0.85	1.025	0.966	0.91	1.025
Female		1.004	0.946	1.065	1.014	0.951	1.081
Male		1.018	0.958	1.081	0.949	0.896	1.004

Average: PM10: 24.4 ug/m3, NO2 15 µg/m3, SO2 = 9.4 µg/m3. Relative risk estimated from Quasi-Poison regression models, adjusting for time trends and seasonal variations, day of week, holiday meteorological factors including temperature and relative humidity. $SO2_{PM10}$, $SO2_{NO2}$ and $SO2_{PM10\ NO2}$ refers to the estimate of SO2 in multiple pollutant models. For cardiovascular disease age 0-14 was excluded from the modelling due to small sample size. For cardiovascular disease age 0-14 was excluded from the modelling due to small sample size.
